# Role of Inhaled Nitric Oxide in the Management of Severe Acute Respiratory Distress Syndrome

**DOI:** 10.3389/fped.2016.00074

**Published:** 2016-08-02

**Authors:** Juliette Lucinda Hunt, Ronald A. Bronicki, Nick Anas

**Affiliations:** ^1^CHOC Children’s Hospital, Orange, CA, USA; ^2^Texas Children’s Hospital, Houston, TX, USA

**Keywords:** pediatric acute respiratory distress syndrome, inhaled nitric oxide, pulmonary hypertension, mechanisms of action, clinical trials

## Abstract

To date, there have been several systematic reviews with meta-analysis that have shown no reduction in mortality with the use of inhaled nitric oxide (iNO) in patients with acute respiratory distress syndrome (ARDS). Importantly, these reports fail to make a distinction between the pediatric and adult patient. The number of adult patients in these reviews are far greater than the number of pediatric patients, which makes it difficult to interpret the data regarding the role of iNO on the pediatric population. Extrapolating data from the adult population to the pediatric population is complicated as we know that physiology and the body’s response to disease can be different between adult and pediatric patients. iNO has been demonstrated to improve outcomes in term and near-term infants with hypoxic respiratory failure associated with pulmonary hypertension. Recently, Bronicki et al. published a prospective randomized control trial investigating the impact of iNO on the pediatric patient population with acute respiratory failure. In this study, a benefit of decreased duration of mechanical ventilation and an increased rate of ECMO-free survival was demonstrated in patients who were randomized to receiving iNO, suggesting that there may be benefit to the use of iNO in pediatric ARDS (PARDS) that has not been demonstrated in adults. iNO has repeatedly been shown to transiently improve oxygenation in all age groups, and yet neonates and pediatric patients have shown improvement in other outcomes that have not been seen in adults. The mechanism that explains improvement with the use of iNO in these patient populations are not well understood but does not appear to be solely a result of sustained improvement in oxygenation. There are physiologic studies that suggest alternative mechanisms for explaining the positive effects of iNO, such as platelet aggregation inhibition and reduction in systemic inflammation. Hence, the role of iNO by various mechanisms and in various age groups warrants further investigation.

## Introduction

In many review articles of acute respiratory distress syndrome (ARDS), the conclusion that the use of iNO does not reduce mortality continues to be perpetuated. Most often, the references for this conclusion are the systematic reviews with meta analysis by Adhikari et al. (1, 2) and Afshari et al. (3) These are well conducted systematic reviews; however, the conclusion made from these studies as it pertains to pediatric ARDS (PARDS) could be challenged.

Analysis of these influential systemic reviews reveals that the number of pediatric studies and overall number of pediatric patients are small. In fact, there are only three pediatric studies on this matter included. The number of pediatric studies are too few to determine the effect of iNO on PARDS outcome.

However, the extrapolation of this adult data to pediatrics has been widely accepted, and there has been inadequate investigation on the subject in pediatrics since these systematic reviews were published. Most have taken for granted that the answer regarding the benefit of iNO in PARDS exists when in fact the question as it applies to pediatrics has never scientifically been evaluated.

The other limitation to understanding the full impact of iNO has been the primary emphasis on its mechanism of action as a selective pulmonary vasodilator in improving V/Q matching and the outcome of mortality. Focusing on the effect of iNO by only this mechanism and this outcome may minimize its therapeutic importance. For example, Nitric oxide (NO) has a variety of effects on the immune system and immune regulation. This could be of particular interest in the context of ARDS, a disease whose pathology is largely a result of the immune response. Some studies in neonates have demonstrated that the incidence of chronic lung disease, a disease that results from an inflammatory process, could be attenuated with the use of iNO (4–6). These findings are important as they give evidence of the effects of iNO that we might be underestimating.

Until we have more pediatric specific data on ARDS and iNO, we are left reviewing what has been learned about ARDS, NO, and iNO from the laboratory and from adults. Equipped with the understanding that the pathology of ARDS and the response to ARDS are likely different between pediatrics and adults and that the mechanisms of iNO are diverse, pediatric practitioners should be challenging the notions that we have accepted from the adult data by re-examining the many properties of iNO and bringing new, important pediatric specific research to the forefront.

## Pathophysiology and Epidemiology of ARDS and PARDS

One of the hallmarks of ARDS is hypoxemia. In this disease state, inflammation leads to injury of the pulmonary capillary endothelium and the alveolar epithelium, which results in permeability and accumulation of polymorphonuclear cells, erythrocytes, platelets, and water in the airspaces (7). Pulmonary edema and inactivation of surfactant result in intrapulmonary shunting and thus hypoxemia.

One of the practice changing results from the ARDS Network study was the reduced mortality in adult patients with ARDS who were randomized to a low tidal volume ventilation strategy (8). The explanation for why mortality is significantly improved with lower tidal volume is not known, but one theory is the reduction in the inflammatory response induced by a lower tidal volume. Ranieri et al. found that patients with ARDS ventilated with a lower tidal volume had a decrease in inflammatory cells and cytokines in their BAL and plasma (9). The association between inflammation and tidal volume, or stretching of the respiratory system, became known as biotrauma (10). This concept emphasizes the significant role the immune system plays in the pathogenesis of ARDS.

Interestingly, although impaired oxygenation is a hallmark of the disease, therapies that have led to improved oxygenation, including iNO, have failed to show a significant reduction in mortality outcome (11). Perhaps the lack of demonstrable mortality benefit is because patients with ARDS often die of multi-system organ failure (MSOF) rather than hypoxemia (12, 13). The presence of MSOF in ARDS is likely due to the host immune response. With damage to the alveolar-capillary barrier, inflammatory cells and cytokines can enter the general circulation and illicit a systemic inflammatory response than can lead to the impairment of multiple organs (14). In this scenerio, the lung becomes the source of inflammation and not solely the target of inflammation.

From an epidemiological standpoint, most studies show that ARDS is less common in children than in adults and that the mortality rate of children is less than adults (15). Sepsis is the most common risk factor in adults with concomitant MSOF as compared to children in whom pulmonary infections are the most common risk factor (16). And perhaps it is the increased MSOF in adults compared to children that explains the difference in mortality risk between these populations. This is an important distinction as we begin to further explore the potential therapeutic benefits of iNO and the possibility that there may be a difference on the impact on adults and children. As a lung-selective therapy, it has been thought that iNO is unlikely to improve overall survival when most adult patients dying from ARDS suffer from multiple organ failure (17). However, since children are more likely to have pneumonia than multi-organ failure, a lung-selective therapy, such as iNO, could provide more of a survival benefit in children than in adults.

Smith and colleagues provide a review of the pathophysiology of ARDS in children and adults (18). The point that the lungs are still developing and growing in children compared to adults is an important distinction emphasized in this paper, particularly because the response to infection and injury are likely different based on the milieu of growth factors, gene expression, and protein expression at various stages of lung development. They also emphasize that the innate immune response is different between children and adults. Infants have impaired clearance of the pathogen and less of the pro inflammatory response, which may be injurious to the lungs. In contrast, adults can clear the pathogen but suffer injury from the inflammatory response itself. The content of surfactant between patients with ARDS and PARDS is also different (19, 20), which may account for difference in benefit in the use of exogenous surfactant between children and adults with ARDS (21) and may also suggest that there is a difference in the injury to the alveolar epithelium between these groups.

Understanding the pathology of ARDS is important as it illustrates potential mechanisms for therapeutic interventions. Additionally, highlighting the differences between ARDS and PARDS, exemplifies that the disease state is not the same in all age groups, and hence the response to therapies, particularly iNO, may be different.

## History of NO and iNO

Since its discovery only a few decades ago, inhaled nitric oxide (iNO) has fascinated many in the field of critical care and medicine in general. Its potential benefits, limited evidence of proven benefit, but ongoing use as a rescue medication continue to be the focus of research and debate.

Nitric oxide gained much notoriety within the scientific community when it was declared the Molecule of the Year by Science in 1992. It was 6 years later that the individuals credited with its discovery, Ferid Murad, Robert Furchgott, and Louis Ignarro were awarded the Nobel prize for Physiology and Medicine in 1998.

In 1993, Rossaint et al. published a landmark article in the New England Journal of Medicine that described the role of iNO in Acute Respiratory Distress Syndrome (ARDS) in reducing pulmonary artery pressures and increasing arterial oxygenation by improving V/Q mismatch without precipitating systemic hypotension (22).

Rossaint’s paper was followed by many important studies including several focused on the neonatal population, which showed definitive benefit of iNO in this patient population. In 1997, the Neonatal Inhaled Nitric Oxide Study Group (NINOS) showed that iNO was beneficial in full-term and nearly full-term infants with hypoxic respiratory failure by demonstrating that the use of iNO reduced the use of extracorporeal membrane oxygenation (ECMO) (23, 24). In 1998, the I-NO/PPHN study group showed that early use of iNO in term infants with persistent pulmonary hypertension (PPHN) had an acute and sustained improvement in oxygenation and a reduction in ECMO use that did not reach statistical significance (25).

In December 1999, The US Food and Drug Administration (FDA) approved the use of INOmax (nitric oxide) for inhalation for the treatment of “term and near-term (>34 weeks) neonates with hypoxic respiratory failure associated with clinical or echocardiographic evidence of pulmonary hypertension where it improves oxygenation and reduces the need for extracorporeal membrane oxygenation” (26). To date, this remains the only licensed indication for the use for iNO.

In August 2000, the American Academy of Pediatrics published a statement on the conditions in which iNO should be used, which endorsed the FDA indications of the use of iNO for newborns with hypoxic respiratory failure (27).

The sole commercial manufacturer of INOmax at the time of FDA approval was INO Therapeutics. In March 2007, INO Therapeutics and Ikaria merged, and INOmax was then exclusively supplied by Ikaria until April of 2015, when Mallinckrodt Pharmaceuticals acquired Ikaria, Inc. Mallinckrodt Pharmaceuticals now maintains the many patents on the INOMAX drug label and INOMAX delivery system, five of which provide protection until 2029 and another five that provide protection until 2031.

## Mechanisms of NO and iNO

Nitric oxide is composed of two atoms that form a gaseous molecule. It is found naturally in the atmosphere and produced in various parts of the body. The enzyme nitric oxide synthase (NOS) converts l-arginine to nitric oxide. There are multiple isoforms of nitric oxide synthase (NOS). nNOS is the neuronal form that is involved in neurotransmission. iNOS is an inducible form involved in the immune response. eNOS is the endothelial nitric oxide synthase isoform, which is the form that is active in healthy vascular endothelial cells (28).

eNOS produces NO that acts specifically on endothelial smooth muscle cells. NO penetrates the cell membrane, which binds to the soluble guanylyl cyclase and activates it forming cGMP. cGMP binds to the cGMP-dependent protein kinase. The activated protein kinase then affixes to ionic channels of the cell membrane and the sarcoplasmic reticulum, which has the effect of decreasing the influx of calcium to the cell, increasing the ejection of calcium from the cell, sequestering the calcium within the sarcoplasmic reticulum, and decreasing calcium mobilization. The net effect of these reactions makes less calcium available for depolarization and contraction, which leads to smooth muscle relaxation (29).


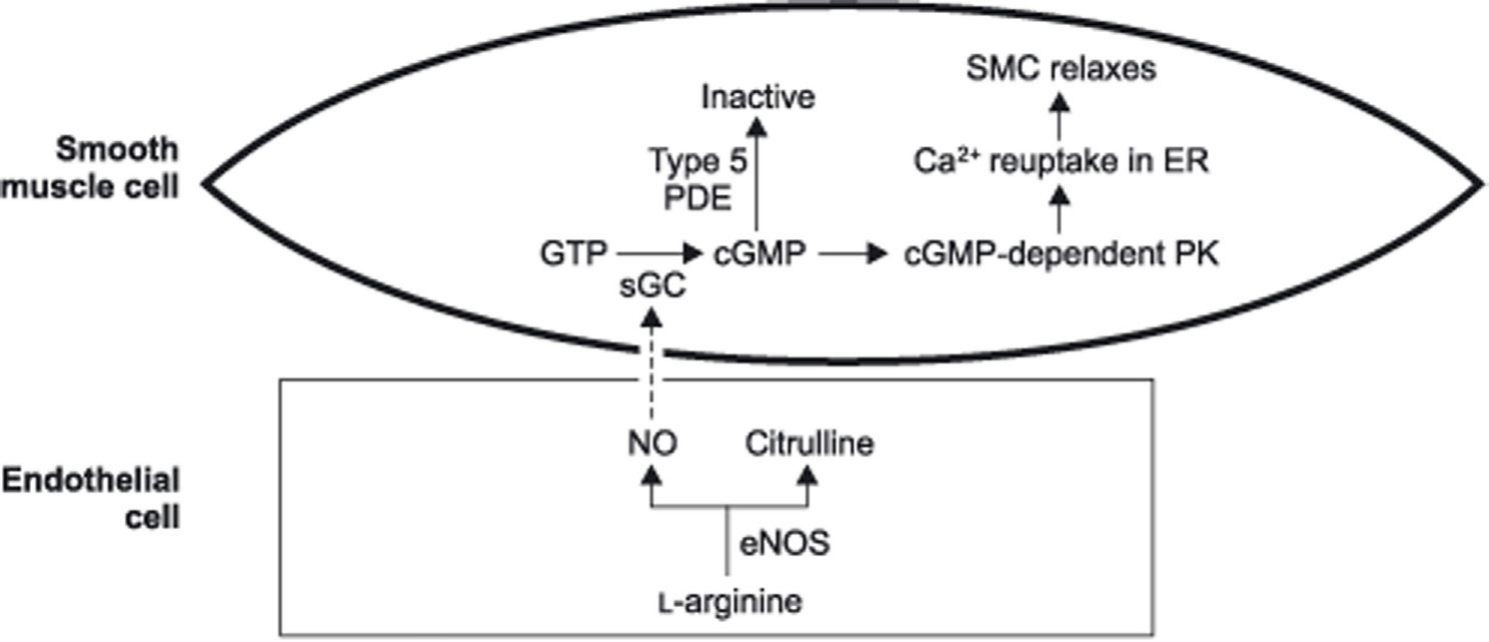


With the understanding of NO’s ability to relax endothelial smooth muscle, multiple laboratory studies were performed to assess the effect of inhaled NO on the pulmonary vasculature (30, 31). In 1991, Frostell et al. demonstrated in lamb models that iNO could produce pulmonary vasodilation by reversing hypoxic pulmonary vasoconstriction or thromboxane-induced pulmonary hypertension without causing systemic hypotension (30). The mechanism similar to that of eNOS is the most likely explanation of how iNO leads to this effect. The inhaled NO is delivered directly to the pulmonary vascular endothelial cells causing the endothelial smooth muscle relaxation of the pulmonary vasculature. The pulmonary vasodilation reduces intrapulmonary shunting when lung disease is present, which allows for increased ventilation/perfusion (V/Q) matching and improved oxygenation.

Nitric oxide is known to have a multitude of other effects on various cells in the body as well. Yet, it is commonly believed that when delivered by inhalation, the effect of nitric oxide is limited to the lungs. As NO is exposed to the bloodstream, it binds rapidly and with a high affinity to hemoglobin, which then inactivates it (32). This is the explanation for why iNO leads to pulmonary vasodilation without causing significant systemic vasodilation or hypotension. It is worth noting, however, that there are some studies to suggest that NO bioactivity may actually reach distant organs even when delivered by inhalation. The implications of this is even less well understood that even further limits our true appreciation of the impact of iNO.

In addition to its effect on endothelial smooth muscle, NO also affects platelet activity. It has been observed *in vitro* that NO activates the guanylate cyclase inside platelets, which increases intraplatelet cGMP. The resulting activation of cGMP-dependent protein kinase here causes a reduction in fibrinogen biding to glycoprotein GP IIb/IIIa, which induces partial inhibition of platelet aggregation (33, 34).

Considering that activation and accumulation of platelets is observed in the alveolar tissues of patients with ARDS (35), and given the effect NO has on platelet aggregation, Samama et al. investigated whether iNO could inhibit platelet aggregation in patients with ARDS (34). They found that the six patients studied showed a significant improvement in oxygenation, significant decrease in pulmonary artery pressure, and a significant decrease in *ex vivo* platelet aggregation after iNO administration without a change in their Ivy bleeding time. They concluded that the improvement in arterial oxygenation and pulmonary circulation are associated with a significant inhibition of platelet aggregation.

Nitric oxide has also been noted to have a number of potentially important effects on the immune system and has been implicated in a variety of mechanisms (36). NO has been shown to alter the balance between T helper (Th)1 and Th2 cells. In particular, NO decreases Th1 proliferation and IL-2 synthesis but increases IL-4 synthesis from Th2 cells. By decreasing Th1 and increasing Th2 responses, NO may inhibit the inflammatory response to viral and bacterial infections. Additionally, NO has an effect on leukocyte adhesion and recruitment to sites of infection. NO has also been reported to have a direct effect on a variety of organisms by directly inhibiting the growth of some viruses, bacteria, parasites, and fungi (37).

As previously stated, ventilator-induced lung injury results in an increase in systemic inflammatory mediators (biotrauma). With the above understanding of the many ways, NO can affect immune regulation and the potential impact of iNO in ARDS is intriguing. Kinsella et al. looked at the effects of iNO on pulmonary hemodynamics, gas exchange, pulmonary edema, and lung myeloperoxidase (MPO) activity, as a marker of lung neutrophil accumulation in extremely premature lambs (38). They studied lambs exposed to iNO and compared them to controls. They found that the lambs exposed to iNO had improved gas exchange but did not have an increase in their lung weight or permeability to albumin after several hours of mechanical ventilation. They also found that the iNO exposed lambs had a reduction in the MPO activity by 79%. They concluded that low dose iNO can augment pulmonary blood flow without worsening pulmonary edema and can decrease lung neutrophil accumulation, which may reduce lung injury. These findings support iNO’s properties as both a pulmonary vasodilator and immune modulator.

In 2000, The Clinical Inhaled Nitric Oxide Research Group (CINRGI) showed that low-dose iNO therapy for PPHN of the newborn could decrease the need for ECMO in these neonates (5). Additionally, this study showed that the group that received low dose iNO also had less chronic lung disease when compared to controls. This work was followed by Schreiber et al. who also demonstrated that treatment with iNO decreased the incidence of chronic lung disease and death among preterm infants with respiratory distress syndrome (6). The finding that chronic lung disease is decreased with the use of iNO, suggests that the benefit of iNO likely extends beyond just pulmonary vasodilation. As chronic lung disease is caused at least in part by irritation and inflammation of the neonatal lungs, the impact of the use of iNO may be a result of iNO’s effect on the immune response to lung injury.

## ARDS and iNO in Adults

As mentioned above, the use of iNO for adult patients with ARDS began in 1993 when Rossaint et al. showed improved oxygenation in this patient population (22). Subsequent studies would also show that oxygenation was improved when iNO was used in adult patients with ARDS but demonstrated that there was no decrease in morbidity and mortality. The lack of survival benefit then called into question the true advantage of using iNO, especially given that in some adults iNO use may increase renal impairment (3, 39).

A number of systematic reviews with meta-analysis have since been conducted to try to answer the question of whether iNO reduces mortality in patients with ARDS. These meta-analysis have looked at available randomized control trials (RCTs) in which there were data on iNO use, ARDS and mortality. In 2007, Adhikari published his first systemic review of 12 RCTs that met inclusion criteria which included a total of 1237 patient with ARDS (1). Of the RCTs included two of them were done with the pediatric population and accounted for only 132 of the total number of patients. The authors of this review found no significant effect of iNO on hospital mortality or number of ventilator-free days. They concluded that iNO had a short lived improvement on oxygenation that conferred no survival benefit. In 2014, Adhikari et al. updated their previous search and analyzed the data from the systemic review to determine if a survival benefit with iNO use could be demonstrated when the patients with ARDS were divided into severe versus mild-moderate ARDS (2). This review had 9 RCTs and 1142 patients that met inclusion criteria. In this review, there were no RCTs with only pediatric patients and only one study that included pediatric patients of which there were only three children. The conclusion from this review was that iNO does not reduce mortality in adults and children with ARDS, regardless of the degree of hypoxemia. Additionally, it should be noted, that many of these trials used a wide range of iNO levels, several of them using 5–10 ppm.

In 2011, Afshari et al. also undertook a systemic review with meta analysis in which they included 14 RCTs with 1303 patients (3). This review included three pediatric studies with a total of 164 pediatric patients. Ten of the studies had a high risk of bias. These authors found no statistically significant effect of iNO on duration of ventilation, ventilator-free days, or on length of stay in the intensive care unit and hospital. They also concluded that iNO results in a transient improvement in oxygenation but does not reduce mortality and may be harmful as it increases the risk of renal impairment in adults.

## ARDS and iNO in Pediatrics

To date, there are no published systemic reviews with meta analysis comprised exclusively of pediatric RCTs investigating the effect of iNO on PARDS. This is not surprising since the findings from the aforementioned systemic reviews reveal a very limited number of eligible pediatric RCTs on the topic. Of those pediatric RCTs that were included, individually they were small in number with study designs that limit the ability to draw conclusions on how iNO impacts the outcome of PARDS.

By including such a small number of pediatric patients in the above systematic reviews with such a larger number of adult studies and much larger number of adult patients it: (1) may diminish the impact and true findings from the pediatric studies and (2) allows the authors to apply conclusions generated largely by adult data to both adult and pediatric patients which may not be valid.

Of the three pediatric RCTs in the aforementioned systemic reviews, only one study was multi-centered which included 7 centers and 108 pediatric patients (40). This multi-center study was a double-blinded, randomized, placebo-controlled trial, which looked at oxygenation as an end-point. The study allowed for cross-over of the placebo group with treatment failure, which made assessment of other endpoints (mortality, duration of ventilation) difficult. The results from this study showed that iNO improved oxygenation at 12 and 24 h, but by 72 h, there was no difference in oxygenation. Interestingly, immunocompromised patients and those patients with more severe oxygenation impairment appeared to have a more sustained improvement in oxygenation.

The other two pediatric RCTs were single-centered studies. The study by Day et al. included 24 pediatric patients and also allowed for cross-over of the placebo group with treatment failure again making a conclusion on outcome differences impossible (41). The third study was conducted by Ibrahim and El-Mohamady and they collected data on 32 pediatric patients for only 24 h to assess the outcome on oxygenation, they did not collect data on other outcomes (42).

The first RCT to evaluate outcomes other than oxygenation in patients with PARDS that received iNO was published in 2014 (43). In this study, Bronicki et al. enrolled 55 pediatric patients in a prospective, randomized placebo-controlled trial that showed a significant reduction in duration of mechanical ventilation and a significantly greater rate of ECMO-free survival. There was a trend toward improved overall 28-day survival that did not meet statistical significance. There was a significant improvement in the oxygenation index of those patient receiving iNO at 12 h, but no difference between the oxygenation index of both groups at 24 h. An important feature of this study is that it suggests that there were benefits to the pediatric patients that received iNO, which did not correlate to a sustained improvement in oxygenation.

The Pediatric Acute Lung Injury Consensus Conference Group was formed in 2012 to better establish the definition for pediatric ARDS and to provide recommendations for management. Their first consensus recommendations for were published in 2015 (44), and the following was their recommendation specifically about iNO and PARDS:

“Inhaled nitric oxide is not recommended for routine use in PARDS. However, its use may be considered in patients with documented pulmonary hypertension or severe right ventricular dysfunction. In addition, it may be considered in severe cases of PARDS as a rescue from or bridge to extracorporeal life support. When used, assessment of benefit must be undertaken promptly and serially to minimize toxicity and to eliminate continued use without established effect. Finally, future study is needed to better define its role, if any, in the treatment of PARDS.” The Group was in strong agreement about this recommendation.

The recommendations by the group indicate that iNO may by considered for severe ARDS but ultimately that more data needs to be collected to understand its role in pediatric ARDS.

## Where Do We Go from Here?

Santschi et al. illustrated that there is wide practice variability in the management in PARDS (45). In the discussion of their paper, they estimated that in order to conduct an international clinical trial to assess a reduction in mortality in PARDS that it would take 60 PICUs and about 4 years to recruit the 800 patients needed. Additionally, RCTs call for the limitation of co-founders that would force us to individually investigate every aspect of management as it pertains to PARDS when the reality is that most patients receive a combination of therapies. The resources it would require to accomplish studies in this fashion are not realistic.

With the challenges of using mortality as an outcome measure in pediatric trials, perhaps alternative outcome metrics in PARDS should be considered and evaluated such as a health related quality of life metric validated in pediatrics, the PedsQL^TM^ Scale (46). As it pertains to the impact of iNO in particular, perhaps we should be looking at how it impacts the Pediatric Multiple Organ Dysfunction Score (P-MODS) (47). As previously stated, ARDS is often not present in isolation and the effects of iNO may spread beyond the lungs in a way that could effect the P-MODS, which could potentially have an additional impact on the PedsQL^TM^ scale.

But likely the most important pursuit is the path toward personalized and precision medicine, an emerging reality with the rapid evolution of genomic medicine. Although ARDS is a disease state with a common histology and pathophysiology, the exact manifestation and the response to therapies, such as iNO, are likely much more individualized than we will ever gather from studying the population as a whole with our tradition approaches. Wong and colleagues have done important work in the field of genomics and septic shock (48). Among his groups, important contributions are the ability for subclass stratification that identifies high risk patients with septic shock based on inflammatory response and genomic profiling. This identification has already been shown to allow for therapy-related clinical decision making particularly as it applies to the use of corticosteroids in septic shock (49). Wong and colleagues have demonstrated a path for similar work that could be done to link individual genomics with ARDS. This could allow for a stratification scheme to identify individuals whose genetic profile might benefit from iNO therapy and the discovery of the true impact of iNO in ARDS might truly be realized. This might prove to be the most worthwhile endeavor to truly understand the impact of iNO.

## Conclusion

As we continue to strive to improve our management of ARDS, it is important to acknowledge that although the disease may look similar from patient to patient that it is likely not the same disease in everyone. The pathology, the pathophysiology and the response to therapy may be quite varied not just by the age of the patient but by other individual characteristics of the patients as well. So although the RCTs and the systemic reviews of iNO and ARDS have helped to guide the management in adults, we should be cautious about extrapolating that data to all patients with ARDS. Perhaps further exploring the impact of iNO on inflammation, coagulation, and applying genomics will provide further insight to the very varied disease of ARDS and the patient populations that are effected by it. The story of iNO, a molecule whose discovery was only within the past 30 years, is still young.

## Author Contributions

JH: majority of original writing. RB: contributed to the outline of the paper with significant direction toward resources used. NA: major editing and some of the original writing.

## Conflict of Interest Statement

The authors declare that the research was conducted in the absence of any commercial or financial relationships that could be construed as a potential conflict of interest.

## References

[1] Adhikari NK Burns KE Friedrich JO Granton JT Cook DJ Meade MO. Effect of nitric oxide on oxygenation and mortality in acute lung injury: systematic review and meta-analysis. BMJ (2007) 334(7597):779. 10.1136/bmj.39139.716794.55 17383982 PMC1852043

[2] Adhikari NK Dellinger RP Lundin S Payen D Vallet B Gerlach H Inhaled nitric oxide does not reduce mortality in patients with acute respiratory distress syndrome regardless of severity: systematic review and meta-analysis. Crit Care Med (2014) 42(2):404–12. 10.1097/CCM.0b013e3182a27909 24132038

[3] Afshari A Brok J Møller AM Wetterslev J. Inhaled nitric oxide for acute respiratory distress syndrome and acute lung injury in adults and children: a systematic review with meta-analysis and trial sequential analysis. Anesth Analg (2011) 112(6):1411–21. 10.1213/ANE.0b013e31820bd185 21372277

[4] Ballard RA Truog WE Cnaan A Martin RJ Ballard PL Merrill JD Inhaled nitric oxide in preterm infants undergoing mechanical ventilation. N Engl J Med (2006) 355:343–53. 10.1056/NEJMoa061088 16870913

[5] Clark RH Kueser TJ Walker MW Southgate WM Huckaby JL Perez JA Low-dose nitric oxide therapy for persistent pulmonary hypertension of the newborn. Clinical Inhaled Nitric Oxide Research Group. N Engl J Med (2000) 342(7):469–74. 10.1056/NEJM200002173420704 10675427

[6] Schreiber MD Gin-Mestan K Marks JD Huo D Lee G Srisuparp P. Inhaled nitric oxide in premature infants with the respiratory distress syndrome. N Engl J Med (2003) 349:2099–107. 10.1056/NEJMoa031154 14645637

[7] Piantadosi CA Schwartz DA The acute respiratory distress syndrome. Ann Intern Med (2004) 141(6):460–70. 10.7326/0003-4819-141-6-200409210-00012 15381520

[8] Network ARDS Ventilation with lower tidal volumes as compared with traditional tidal volumes for acute lung injury and the acute respiratory distress syndrome. N Engl J Med (2000) 342:1301–8. 10.1056/NEJM200005043421801 10793162

[9] Ranieri VM Suter PM Tortorella C De Tullio R Dayer JM Brienza A Effect of mechanical ventilation on inflammatory mediators in patients with acute respiratory distress syndrome: a randomized controlled trial. JAMA (1999) 282(1):54–61. 10.1001/jama.282.1.54 10404912

[10] Tremblay LN Slutsky AS Ventilator-induced lung injury: from barotraumas to biotrauma. Proc Assoc Am Physicians (1998) 110:482–8. 9824530

[11] Gattinoni L Tognoni G Pesenti A Taccone P Mascheroni D Labarta V Effect of prone positioning on the survival of patients with acute respiratory failure. N Engl J Med (2001) 345(8):568–73. 10.1056/NEJMoa010043 11529210

[12] Doyle RL Szaflarski N Modin GW Wiener-Kronish JP Matthay MA. Identification of patients with acute lung injury. Predictors of mortality. Am J Respir Crit Care Med (1995) 152(6 Pt 1):1818–24. 10.1164/ajrccm.152.6.8520742 8520742

[13] Montgomery AB Stager MA Carrico CJ Hudson LD. Causes of mortality in patients with the adult respiratory distress syndrome. Am Rev Respir Dis (1985) 132(3):485–9. 4037521 10.1164/arrd.1985.132.3.485

[14] Slutsky AS Tremblay LN Multiple system organ failure. Am J Respir Crit Care Med (1998) 157(6):1721–5. 10.1164/ajrccm.157.6.9709092 9620897

[15] Zimmerman JJ Akhtar SR Caldwell E Rubenfeld GD. Incidence and outcomes of pediatric acute lung injury. Pediatrics (2009) 124(1):87–95. 10.1542/peds.2007-2462 19564287

[16] Rubenfeld GD Caldwell E Peabody E Prevalence and outcomes of acute lung injury. N Engl J Med (2005) 353:1685–93. 10.1056/NEJMoa050333 16236739

[17] Ichinose F Roberts JD Jr Zapol WM Inhaled nitric oxide: a selective pulmonary vasodilator: current uses and therapeutic potential. Circulation (2004) 109:3106–11. 10.1161/01.CIR.0000134595.80170.62 15226227

[18] Smith LS Zimmerman JJ Martin TR. Mechanisms of acute respiratory distress syndrome in children and adults: a review and suggestions for future research. Pediatr Crit Care Med (2013) 14(6):631–43. 10.1097/PCC.0b013e318291753f 23823199

[19] Greene KE Wright JR Steinberg KP Ruzinski JT Caldwell E Wong WB Serial changes in surfactant-associated proteins in lung and serum before and after onset of ARDS. Am J Respir Crit Care Med (1999) 160(6):1843–50. 10.1164/ajrccm.160.6.9901117 10588595

[20] LeVine AM Lotze A Stanley S Stroud C O’Donnell R Whitsett J Surfactant content in children with inflammatory lung disease. Crit Care Med (1996) 24(6):1062–7. 10.1097/00003246-199606000-00029 8681574

[21] Duffett M Choong K Ng V Randolph A Cook DJ. Surfactant therapy for acute respiratory failure in children: a systematic review and meta-analysis. Crit Care (2007) 11(3):R66. 10.1186/cc5944 17573963 PMC2206432

[22] Rossaint R Falke KJ Lopez F Slama K Pison U Zapol WM Inhaled nitric oxide for the adult respiratory distress syndrome. N Engl J Med (1993) 328:399–405. 10.1056/NEJM199302113280605 8357359

[23] Neonatal Inhaled Nitric Oxide Study Group. Inhaled nitric oxide in full-term and nearly full-term infants with hypoxic respiratory failure. N Engl J Med (1997) 336:597–604. 10.1056/NEJM199702273360901 9036320

[24] Roberts JD Jr Fineman JR Morin FC Shaul PW Rimar S Schreiber MD Inhaled nitric oxide and persistent pulmonary hypertension of the newborn. N Engl J Med (1997) 336:605–10. 10.1056/NEJM199702273360902 9032045

[25] Davidson D Barefield ES Kattwinkel J Dudell G Damask M Straube R Inhaled nitric oxide for the early treatment of persistent pulmonary hypertension of the term newborn: a randomized, double-masked, placebo-controlled, dose-response, multicenter study. The I-NO/PPHN Study Group. Pediatrics (1998) 101(3 Pt 1):325–34. 10.1542/peds.101.3.325 9480993

[26] Approval Letter by US Food and Drug Administration on the FDA website. Available from: http://www.accessdata.fda.gov/drugsatfda_docs/nda/99/20845_INOmax_Approv.pdf

[27] American Academy of Pediatrics Committee on Fetus and Newborn. Use of inhaled nitric oxide. Pediatrics (2000) 106(2 Pt 1):344–5. 10.1542/peds.106.2.344 10920164

[28] Alderton WK Cooper CE Knowles RG. Nitric oxide synthases: structure, function and inhibition. Biochem J (2001) 357(Pt 3):593–615. 10.1042/0264-6021:3570593 11463332 PMC1221991

[29] Katz I Inhaled Nitric Oxide: Therapeutic Uses And Potential Hazards. Glenview, IL: PCCSU (Vol. 25, Lesson 22) (2012).

[30] Frostell C Fratacci MD Wain JC Jones R Zapol WM Inhaled nitric oxide. A selective pulmonary vasodilator reversing hypoxic pulmonary vasoconstriction [published correction appears in Circulation. 1991;84: 2212]. Circulation (1991) 83:2038–47. 10.1161/01.CIR.83.6.2038 2040056

[31] Roberts JD Jr Chen TY Kawai N Wain J Dupuy P Shimouchi A Inhaled nitric oxide reverses pulmonary vasoconstriction in the hypoxic and acidotic newborn lamb. Circ Res (1993) 72:246–54. 10.1161/01.RES.72.2.246 8380356

[32] Joshi MS Ferguson TB Jr Han TH Hyduke DR Liao JC Rassaf T Nitric oxide is consumed, rather than conserved, by reaction with oxyhemoglobin under physiological conditions. Proc Natl Acad Sci U S A (2002) 99(16):10341–6. 10.1073/pnas.152149699 12124398 PMC124916

[33] Radomski MW Moncada S Regulation of vascular homeostasis by nitric oxide. Thromb Haemost (1993) 70(1):36–41. 7694388

[34] Samama CM Diaby M Fellahi JL Mdhafar A Eyraud D Arock M Inhibition of platelet aggregation by inhaled nitric oxide in patients with acute respiratory distress syndrome. Anesthesiology (1995) 83(1):56–65. 10.1097/00000542-199507000-00007 7605019

[35] Idell S Maunder R Fein AM Switalska HI Tuszynski GP McLarty J Platelet-specific alpha-granule proteins and thrombospondin in bronchoalveolar lavage in the adult respiratory distress syndrome. Chest (1989) 96:1125–32. 10.1378/chest.96.5.1125 2530064

[36] Mannick JB Immunoregulatory and antimicrobial effects of nitrogen oxides. Proc Am Thorac Soc (2006) 3:161–5. 10.1513/pats.200505-048BG 16565425 PMC2658681

[37] Bogdan C. Nitric oxide and the immune response. Nat Immunol (2001) 2:907–16. 10.1038/ni1001-907 11577346

[38] Kinsella JP Parker TA Galan H Sheridan BC Halbower AC Abman SH Effects of inhaled nitric oxide on pulmonary edema and lung neutrophil accumulation in severe experimental hyaline membrane disease. Pediatr Res (1997) 41:457–63. 10.1203/00006450-199704000-00002 9098845

[39] Ruan S-Y Huang T-M Wu H-Y Wu H-D Yu C-J Lai M-S. Inhaled nitric oxide therapy and risk of renal dysfunction: a systematic review and meta-analysis of randomized trials. Crit Care (2015) 19(1):137. 10.1186/s13054-015-0880-2 25887847 PMC4384233

[40] Dobyns EL Cornfield DN Anas NG Fortenberry JD Tasker RC Lynch A Multicenter randomized controlled trial of the effects of inhaled nitric oxide therapy on gas exchange in children with acute hypoxemic respiratory failure. J Pediatr (1999) 134:406–12. 10.1016/S0022-3476(99)70196-4 10190913

[41] Day RW Allen EM Witte MK A randomized, controlled study of the 1-hour and 24-hour effects of inhaled nitric oxide therapy in children with acute hypoxemic respiratory failure. Chest (1997) 112(5):1324–31. 10.1378/chest.112.5.1324 9367476

[42] Ibrahim TS El-Mohamady HS Inhaled nitric oxide and prone position: how far they can improve oxygenation in pediatric patients with acute respiratory distress syndrome? J Med Sci (2007) 7:390–5. 10.3923/jms.2007.390.395

[43] Bronicki RA Fortenberry J Schreiber M Checchia PA Anas NG. Multicenter randomized controlled trial of inhaled nitric oxide for pediatric acute respiratory distress syndrome. J Pediatr (2015) 166(2):365–9.e1. 10.1016/j.jpeds.2014.10.011 25454942

[44] Pediatric Acute Lung Injury Consensus Conference Group. Pediatric acute respiratory distress syndrome: consensus recommendations from the Pediatric Acute Lung Injury Consensus Conference. Pediatr Crit Care Med (2015) 16(5):428–39. 10.1097/PCC.0000000000000350 25647235 PMC5253180

[45] Santschi M Jouvet P Leclerc F Gauvin F Newth CJ Carroll CL Acute lung injury in children: therapeutic practice and feasibility of international clinical trials. Pediatr Crit Care Med (2010) 11(6):681–9. 10.1097/PCC.0b013e3181d904c0 20228688

[46] Varni JW Seid M Kurtin P PedsQL^TM^ 4.0: reliability and validity of the Pediatric Quality of Life Inventory^TM^ Version 4.0 Generic Core Scales in healthy and patient populations. Med Care (2001) 39(8):800–12. 10.1097/00005650-200108000-00006 11468499

[47] Graciano AL Balko JA Rahn DS Ahmad N Giroir BP. The Pediatric Multiple Organ Dysfunction Score (P-MODS): development and validation of an objective scale to measure the severity of multiple organ dysfunction in critically ill children. Crit Care Med (2005) 33(7):1484–91. 10.1097/01.CCM.0000170943.23633.47 16003052

[48] Wong HR. Genome-wide expression profiling in pediatric septic shock. Pediatr Res (2013) 73(4 Pt 2):564–9. 10.1038/pr.2013.11 23329198 PMC3615026

[49] Wong HR Cvijanovich NZ Anas N Allen GL Thomas NJ Bigham MT Developing a clinically feasible personalized medicine approach to pediatric septic shock. Am J Respir Crit Care Med (2015) 191(3):309–15. 10.1164/rccm.201410-1864OC 25489881 PMC4351580

